# Co‐Designing End‐of‐Life Care for People With Pre‐Existing Severe Mental Illness: Insights From Multiple Stakeholder Consultations

**DOI:** 10.1111/inm.70226

**Published:** 2026-01-28

**Authors:** Jialiang Cui, Cong Zheng, Cheryl Chi‐Yan Yeung, Helen Yue‐lai Chan

**Affiliations:** ^1^ Department of Social Work The Chinese University of Hong Kong Hong Kong SAR China; ^2^ The Nethersole School of Nursing, Faculty of Medicine The Chinese University of Hong Kong Hong Kong SAR China

## Abstract

People with severe mental illness (SMI) experience significant health disparities, and their end‐of‐life care remains underdeveloped. Employing a co‐design approach, this study engaged a range of stakeholders in Hong Kong, including people with SMI, their family members and professionals in mental health and palliative care, to identify service gaps, challenges and opportunities for improving end‐of‐life care for this population. Findings highlighted the suboptimal state of end‐of‐life care for people with SMI and the underlying challenges at individual, organisational and community levels, including limited knowledge of end‐of‐life care, low help‐seeking motivation, communication difficulties, limited support networks, insufficient on‐site medical support for end‐of‐life care, unclear guidelines for dying‐in‐place, inadequate collaboration between medical and social sectors, mental health stigma, insufficient legal support and cultural taboo surrounding death. Recommendations were co‐developed with participants to inform the design of person‐centred, contextually responsive models that promote equitable and dignified end‐of‐life care for people with SMI.

## 1 Introduction

Compared to the general population, people with severe mental illness (SMI), such as schizophrenia, bipolar disorder and major depression, face substantial health disparities. Studies have revealed a significantly shortened survival rate for those with SMI, emphasising a higher incidence and prevalence of morbidity and mortality (Coffey et al. 2025; Hannigan et al. 2022; Firth et al. 2019). The mortality gap among those with SMI has been extensively studied, uncovering multifactorial causes. Most notably, serious illnesses such as cancer and chronic obstructive pulmonary disease contribute significantly to this increased mortality (Shalev et al. 2020). These figures underscore the urgent need for specialised care and attention to this underserved population, particularly in end‐of‐life care.

Despite this pressing need, end‐of‐life care for people with SMI remains underdeveloped and often suboptimal. This group is less likely to receive specialist palliative care and high‐intensity treatment at the end of life (Coffey et al. 2025; Videbech et al. 2025). Care delivery is often complicated by multiple challenges. At the patient level, the complexity of illnesses, co‐occurring conditions, limited self‐care capacity, and mental health stigma experienced by individuals with SMI can significantly hinder timely and appropriate access to palliative and end‐of‐life care (Bentson et al. 2023; Hannigan et al. 2022). For instance, patients with SMI may exhibit atypical symptom presentation or reluctance toward screening and treatment, potentially due to psychiatric symptoms or prior negative experiences with the health system (Berk et al. 2012; Evenblij et al. 2016). This may affect the timely diagnosis and management of life‐limiting conditions such as cancer or heart disease.

Further, challenges also lie in interprofessional knowledge‐building and collaboration between palliative and mental health care. Professionals across these domains often lack shared knowledge, training, and experience to deliver coordinated care for individuals with both mental health and palliative care needs (Evenblij et al. 2016; Shalev et al. 2025). For example, some palliative care professionals may feel insecure when discussing SMI, which can result in less openness during such conversations (Bøndergaard et al. 2025). Further, complex interactions between medications for physical and mental conditions may be overlooked without collaborative monitoring, increasing the risk of adverse effects or complications (Bentson et al. 2023). The absence of an effective referral mechanism, particularly for individuals with psychosis, may leave staff unaware of critical information necessary for accurate diagnosis and assessment (Jerwood et al. 2018). In addition, discrimination against patients with psychiatric conditions was observed within the palliative care system, leading to selective care provision (Shalev et al. 2025).

Collectively, these challenges highlight the pressing need to develop a holistic, person‐centred end‐of‐life care model—one that is specifically tailored to individuals with SMI and grounded in interprofessional training, cross‐sectoral collaboration, inclusive service design, and the realities of the local context in which care is delivered. Despite increasing attention in recent years, there remains a lack of active involvement of multiple stakeholders—especially individuals with SMI—in the review and refinement of services.

### 1.1 Current Study

This study was conducted in Hong Kong with the aim of synthesising the perspectives of multiple stakeholders on developing the end‐of‐life care of people with SMI. In recent years, end‐of‐life care in Hong Kong has made notable progress. Since 2016, the Jockey Club End‐of‐Life Community Care Project (JCECC), sponsored by a local philanthropic organisation, The Hong Kong Jockey Club Charities Trust, has been working to improve the quality of end‐of‐life care, enhance the capacity of service providers and raise public awareness about end‐of‐life issues (JCECC 2016). The project has developed a care model that supports the identification of older adults with life‐limiting illness, assessment (informed primarily by the Integrated Palliative Outcome Scale; Murtagh et al. 2019), and intervention—addressing their physical, psycho‐social‐spiritual and practical needs.

Following this initiative, relevant policies and services proliferated in the older population over the past decade. This has given rise to growing attention to the end‐of‐life care needs of people with SMI, due to the unique challenges faced by this group, as previously discussed, which may further complicate the delivery of care. This highlights the paramount importance of making adjustments to policies and services that are specifically tailored to this group. In response to this service gap, JCECC: Unison was launched in 2024. Drawing on the lessons of JCECC, the project expands to deliver individualised palliative and end‐of‐life care to people with disabilities, as well as their informal and formal supporters. At this initial stage, the project teams serving people with SMI mainly target existing users of residential, vocational and community services within the local mental health system.

Using a co‐design approach, this study engaged a diverse range of stakeholders—including people with SMI, their family member, and professionals in mental health and palliative care—to identify service gaps, practical challenges and potential adaptations needed to develop a responsive care model for people with SMI.

## 2 Methods

### 2.1 Study Design

This study was conducted between August 2024 and May 2025 employing a co‐design method. Co‐design is recognised as an approach that can positively contribute to service improvement and empower service users by involving them, along with providers, researchers and other stakeholders, in the service design process (Slattery et al. 2020). It has been implemented in practice research to improve mental health services (Hawke et al. 2024), palliative care (Blackwell et al. 2017) and palliative and end‐of‐life care for people with SMI (Jerwood 2019).

The co‐design approach consists of three phases to systematically gather information by integrating a literature review, clinical observations and consensus workshops. First, a literature review was conducted to examine and synthesise both local and international knowledge regarding service gaps and existing strategies for organising palliative and end‐of‐life care for people with SMI.

In the second phase, on‐site consultations were carried out with three clinical teams of JCECC: Unison, which provided end‐of‐life care for people with SMI. During these visits, the working environment and activities were observed, and preliminary insights were gathered from practitioners on the challenges, service gaps and potential improvements in palliative and end‐of‐life care based on their frontline experiences.

In the third phase, drawing on insights from the previous steps, two research‐to‐practice co‐design workshops were conducted to gather views from a broader group of stakeholders to inform need identification and model adjustments. This kind of consensus workshop has been acknowledged as an effective approach to bridging the gap between research and practice by facilitating the engagement of diverse stakeholders and enabling the elicitation of insights that inform the development of service models (Pillemer et al. 2015; Trevino et al. 2020).

Ethical approval was granted by the Survey and Behavioural Research Ethics Committee of the Chinese University of Hong Kong (Reference No. SBRE‐24‐0503).

### 2.2 Participants

For co‐design workshops, the research team distributed recruitment information with the support of the clinical teams of JCECC: Unison, self‐help groups of people with SMI, and other major mental health organisations in Hong Kong. The inclusion criteria for participants in the two consensus workshops were: (1) people with SMI, their family members, or staff providing health and social care to people with SMI; (2) an interest in topics related to palliative and end‐of‐life care; (3) aged 18 or older; and (4) willing to participate voluntarily with the capacity to provide informed consent. In total, 38 participants took part in the two co‐design workshops, comprising individuals with SMI and their family members, as well as social workers and nurses from major mental health organisations.

### 2.3 Data Collection and Analysis

Drawing on insights from the literature review and on‐site consultations, the two co‐design workshops were conducted in March and April 2025. The first workshop, which included mostly practitioners working in or collaborating with residential services (*n* = 15), focused primarily on the experiences of people with SMI residing in mental health residential services (i.e., non‐medical, community‐based facilities providing housing and support). The second workshop, which involved individuals with SMI, a caregiver, and practitioners living or working in community settings (*n* = 23), concentrated on the experiences of those living independently in the community. In total, 38 participants joined the two co‐design workshops, including 26 professionals from mental health organisations (21 social workers and five nurses), 11 people with SMI, and one family caregiver. Notably, six of the social workers in the two workshops also held managerial or supervisory roles while providing direct services. Their involvement enriched the discussions by offering diverse perspectives and fostering consensus through the integration of multiple viewpoints. Each workshop lasted approximately 3 hours.

**FIGURE 1:**
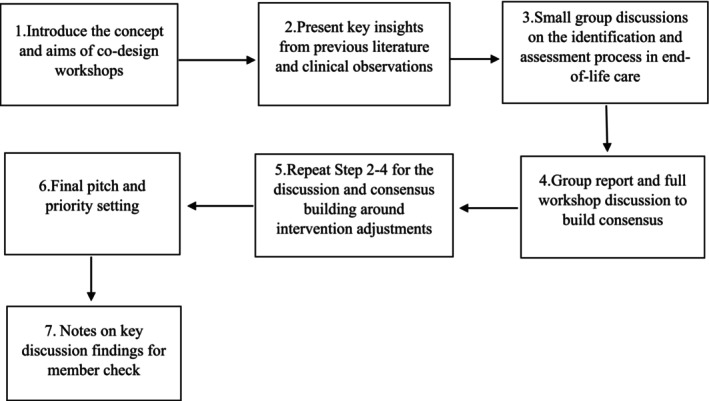
An overview of the co‐design discussion process.

As shown in Figure 1, the co‐design workshops followed a structured discussion process. At the beginning, the objectives of the workshop were clearly explained to participants to help them understand how they could contribute. The subsequent discussion was organised into small groups, with four groups in each workshop. The structure of the discussion was informed by the original end‐of‐life care model for elderly individuals, which served as a baseline model for the JCECC: Unison teams. This model mainly consists of three phases: identification, assessment, and interventions.

First, the facilitator focused on the identification and assessment phases by presenting key insights from previous literature and clinical observations, followed by small group discussions on relevant issues and required adjustments, and then a full‐group discussion for feedback and consensus building. Each small group discussion was facilitated by 1–2 research team members. The same process was then repeated to explore issues related to intervention approaches for end‐of‐life care for people with SMI. The workshop concluded with an overview of the discussion and a final pitch for setting action priorities.

To create a safe and comfortable environment for participants to share their perspectives, and considering the difficulty of capturing voices when five group discussions were conducted simultaneously in one room, the discussions were not audio‐recorded. Instead, a research team member in each small group took detailed notes on a computer during the sessions. The accuracy of these notes was subsequently verified through summaries of the key discussion points presented by each group during the sharing session.

Thematic analysis, following the approach developed by Braun and Clarke (2013), was employed to analyse the notes. An open‐coding process was applied to the notes from each round of small‐group and full workshop discussions. These codes were then developed into overarching themes, incorporating triangulated insights from on‐site observations. Three research team members independently reviewed these materials and collaboratively refined and consolidated the themes through an iterative process, ensuring clarity, coherence, and alignment with the study's objectives. To enhance trustworthiness, member check was conducted by sending summary notes of key discussion findings to workshop participants who had agreed to provide contact details. The thematic analysis was guided by two research team members with extensive experience in this approach.

## 3 Findings

The analysis revealed a constellation of personal, professional, and structural factors that influence the quality of care experienced by people with SMI in their final stages of life. Synthesising multiple stakeholder perspectives, this section first outlines perceptions of the current state of end‐of‐life care for people with SMI in Hong Kong. It then presents key themes on needs and challenges underpinning the service gaps at the individual, organisational, and community levels. Accordingly, the section concludes with recommended adjustments, collectively identified by stakeholders during the co‐design workshops.

### 3.1 Present Situation of End‐of‐Life Care for People With SMI

When these co‐design workshops were held, JCECC: Unison had been piloted for around half a year. Although participants acknowledged its promise, they described the broader end‐of‐life care system for people with SMI as still suboptimal. The period between the identification of a terminal illness and death is often brief, which many described as a ‘sudden death’. This was acutely felt in both community and residential settings, with many participants—particularly social workers—sharing cases where service users were diagnosed with terminal illnesses only after acute deterioration, limiting the time or opportunity for appropriate end‐of‐life care and support.

In addition to delays in the timely identification of terminal illness, participants observed a general neglect of, or lack of sensitivity to, the medical care needs of people with SMI. One caregiver described how a service user who developed persistent digestive issues was told ‘to be observed a little longer’, which ultimately led to an advanced stage of a serious disease. Service users themselves also reported that their physical complaints were frequently downplayed or misattributed to psychiatric conditions. A nurse in a residential setting recalled a resident's chest pain being dismissed until it escalated into an emergency.

### 3.2 The Challenges Underpinning the Service Gaps

#### 3.2.1 Individual‐Level Barriers

Discussing the factors underlying the current suboptimal care provision, participants raised a range of issues related to the needs and challenges faced by individuals with SMI and their caregivers, including limited knowledge of end‐of‐life care and low help‐seeking motivation, communication difficulties in assessment, as well as limited support networks.

Limited knowledge of end‐of‐life care and help‐seeking motivation emerged as a key challenge identified by participants. It was observed that some service users and their family members have a limited understanding of terminal illnesses and their related symptoms, as well as end‐of‐life services such as advance care planning or palliative care. Even among those with some awareness, the motivation to engage in deeper conversations can be minimal. As one social worker noted, the service user did not like to talk about end‐of‐life—maybe just said one or two sentences—making it difficult to gain deeper insight into their thoughts. Some practitioner participants attributed this indifference to the negative symptoms of SMI, which they believed led patients to feel that ‘nothing really matters’. Other service‐user participants pointed to prior negative experiences with the health system (e.g., involuntary hospitalisation), which may contribute to reluctance in seeking end‐of‐life care.

Difficulties in communication were also frequently highlighted by practitioners, even when some motivation for engagement was noted. Many participants attributed this to the (temporary) cognitive difficulties experienced by some clients with SMI, arising from psychiatric symptoms, side effects of medication or ageing effects. This may also be related to the effects of dementia, as people with SMI have a significantly increased risk of developing dementia (Stevenson‐Hoare et al. 2024). For example, a nurse shared a case in which it was almost impossible to have an in‐depth discussion with a patient who was often drowsy due to high doses of medication. As a result, the current format of the palliative care outcome assessment tool used in the project was considered by many practitioners as inadequate and impractical for capturing the holistic needs of people with SMI. For example, certain wordings related to psychological and spiritual needs (e.g., whether feeling good as a person or life as worthwhile) were reported as confusing for users with cognitive difficulties and challenging for staff to assess. In addition, some participants reported that patients' responses are often inconsistent in both content and level of detail, creating challenges in formulating effective and consistent care plans.

A limited support network was emphasised as another critical factor. Unlike people with other types of disabilities, those with SMI often have fewer strong and positive relationships with family and friends. Many individuals with early‐onset SMI are less likely to marry or be supported by spouses and children as they age. Participants observed that a large proportion of this group live in residential settings or alone in the community, receiving only short and infrequent visits from relatives. This lack of consistent support undermines the quality of end‐of‐life care in many ways. Without strong and timely family involvement, service users are at greater risk of delayed diagnosis. Participants also noted that, without such support, people with SMI often lack the financial and practical resources needed to discuss an advance care plan and sign legally recognised end‐of‐life documents. In addition, practitioner participants further described the impact of this challenge in assessment and intervention. Without family participation in the assessment process, professionals may be unable to form a holistic and accurate understanding of the person's end‐of‐life care needs. In some cases, family members—particularly those who have endured a traumatic relationship with their relative with SMI—are less likely to be engaged in end‐of‐life care for the patient, making certain interventions more challenging and less effective.

### 3.3 Organisational‐Level Barriers

At the organisational level, participants commonly expressed that mental health staff, whether in residential or community settings, are generally unprepared to deliver end‐of‐life care.

A lack of medical support for end‐of‐life care within these settings was identified as a key factor contributing to this perception. Most facilities are staffed primarily by psychiatric nurses, who are often not familiar with palliative care. Aside from insufficient medical support within the setting, outreach services offered by visiting doctors were also regarded as inadequate to support end‐of‐life care. Unlike residents of elderly care homes who can access outreach multi‐disciplinary services—including medical assessments, care management and rehabilitation programs—through the government's Community Geriatric Assessment Team, residents with SMI have difficulty obtaining comparable services. Consequently, serious or life‐threatening conditions may not be prevented, diagnosed or treated in a timely manner.

Unclear guidelines for ‘dying in place’ were identified as another critical challenge. In residential care in particular, although recent legislative amendments have been made to facilitate the choice of dying in place (The Government of the Hong Kong Special Administrative Region 2024), many participants described feeling at a loss about how to implement it. This uncertainty stems not only from a relative unfamiliarity with end‐of‐life care, but also from the absence of clear internal protocols and appropriate facilities. For instance, most care homes for people with SMI do not have dedicated spaces for end‐of‐life care. Even in homes that are developing such rooms, confusion and hesitation persist over when a resident should be moved there and how existing staff should be mobilised to provide support.

Inadequate collaboration between the medical and social sectors was also raised as a significant barrier. It was observed that professionals often had a limited understanding of each other's roles, available services and procedures. Participants also observed that many palliative care or oncology professionals might be unfamiliar with how to communicate effectively with people with SMI, undermining both early identification and the patient's care experience. The absence of a formal referral mechanism by the JCECC: Unison clinical teams to palliative care often forces patients to move between multiple hospital departments, including emergency units and government clinics, placing a heavy burden on their already frail health and increasing the strain on family caregivers and social service staff who accompany them.

Taken together, one example encapsulates these issues: as a senior nurse commented, even when a terminal diagnosis is made, unclear and uncoordinated workflows, weak coordination with hospitals and limited on‐site medical support often result in residents being sent repeatedly to emergency departments. These gaps can seriously compromise their quality of life at the end of life.

### 3.4 Community‐Level Barriers

At a broader community level, participants highlighted the absence of a supportive sociocultural climate and environment for ensuring equitable and dignified care for people with SMI during their final stages of life.

Mental health stigma was identified as the most prominent underlying factor. This includes not only overt prejudice but also more subtle forms of discrimination and exclusion that hinder access to services. One participant with SMI shared that she often encountered difficulties when requesting home care services; some staff, upon learning of her mental health history, assumed she was ‘aggressive’ and refused to provide assistance. Within the health care system, some medical personnel were reported to hold stereotypes about people with SMI—believing they universally lack mental capacity and are therefore unable to make decisions about their medical or care plans during serious illness.

Insufficient legal support was considered a related barrier. Participants expressed serious concerns about the legal frameworks governing advance directives, wills and enduring powers of attorney. Although the law in Hong Kong does not prohibit people who are considered as mentally incapacitated, which include people with SMI, from signing such documents, uncertainty around mental capacity assessment and limited access to legal assistance leave them vulnerable. A social worker participant explained that even when a resident wished to appoint an enduring power of attorney, the facility had no established procedures and legal aid was unavailable. A community nurse cited a case in which an advance directive completed by a patient with SMI was disregarded by emergency department staff, who questioned its validity. Without these legal instruments, people with SMI risk losing autonomy over their bodies and assets during the palliative and end‐of‐life stages, and after death, advance care plans made beforehand may ultimately fail to be implemented under legal protection.

Cultural taboos surrounding death further complicate the delivery of end‐of‐life care. In the local context, discussing death is often considered unsettling. A participant with SMI and late‐stage cancer shared that she deliberately avoided talking about the topic with her family, as it made everyone feel uncomfortable. This cultural unease also affects the implementation of end‐of‐life care within services. For example, participants noted that resistance and objections from other residents or their families often arose when introducing end‐of‐life care services and facilities in residential homes. In addition, mental health stigma adds another layer to this taboo. When clients attempted to discuss death‐related issues, their intentions were often misinterpreted as suicidal ideation. One service user recounted that simply mentioning the word ‘will’ during a counselling session triggered an alarm, leading to unnecessary risk assessments. Such practices can deepen the internalisation of mental health stigma, discouraging individuals from reporting severe symptoms and reducing their willingness to actively engage in advance care planning.

### 3.5 Recommendations

In the workshops, following in‐depth discussions on the needs and challenges underpinning service gaps, participants were invited to share their insights and practical wisdom on how these barriers might be overcome. They proposed a range of adjustments to current practice that address the multi‐level challenges identified across individual, organisational, and community spheres, with the aim of advancing end‐of‐life care for people with SMI. Incorporating insights gathered during stage‐2 consultations, these recommendations are summarised below and illustrated in Table 1.

**TABLE 1:** Key challenges and recommendations in providing end‐of‐life care for people with SMI.

	Significant challenges	Recommendations
Individual level	Limited knowledge of end‐of‐life care Low help‐seeking motivation Communication difficulties Limited support networks	Develop appropriate assessment tools/methods Develop disability‐friendly advance care planning tools Provide educational programs for people with SMI and their supporters Offer informational and financial support for low‐income families Provide support for post‐death arrangements for people with SMI without family support
Organisational level	Insufficient medical support for end‐of‐life care Inadequate collaboration between the medical and social sectors Unclear guidelines for dying in place	Improve referral and information‐sharing mechanisms Provide training for palliative care staff on communication and the rights of people with SMI Provide training and resource kits to mental health workers on palliative and end‐of‐life care Clarify guidelines for dying in place and share case examples Enhancing medical support through visiting palliative care specialists in psychiatric settings
Community level	Mental health stigma Cultural taboo around death Insufficient legal support	Provide clear guidance (with casebook) for signing legal documents Promote public education to combat stigma and taboos Promote supported decision‐making frameworks Mobilise community involvement through volunteer training/services

To address personal‐level barriers, participants emphasised the need for appropriate assessment tools and end‐of‐life resources that accommodate cognitive fluctuations, communication difficulties, and varying levels of health literacy among people with SMI. Suggestions included developing disability‐friendly needs assessment and advance care planning tools—such as incorporating visual aids, simplified language, and step‐by‐step guides for those with cognitive challenges. Participants with SMI who did not face such limitations recommended greater flexibility to include more rigorous and comprehensive legal and medical information, noting that an overreliance on visual aids or cartoon‐style formats could feel infantilising. Furthermore, tailored educational programs (e.g., information on terminal illness and palliative care) for people with SMI and their supporters were recommended in community and residential settings. Lastly, participants called for targeted practical support for people with SMI, particularly low‐income individuals, to complete legally recognised documents (e.g., advance directives, wills and enduring powers of attorney) and to arrange post‐death matters where family networks are absent.

At the service‐provision level, participants recommended strengthening referral and information‐sharing mechanisms between mental health and palliative care services. Reciprocal training with specialised resource kits was proposed so that mental health professionals are equipped with palliative care principles and communication skills, while palliative care professionals gain competence in working with people with SMI. Clear, practical guidelines for dying in place, accompanied by case examples, were urged to reduce uncertainty and improve care quality. To address gaps in medical support, participants suggested allocating funding to involve visiting staff with expertise in palliative care for psychiatric settings, enabling residents who wish to die in place to do so with appropriate clinical oversight.

At the community level, participants highlighted the need for legal support, targeted training, and sustained public education to combat mental health stigma and taboo surrounding death. They called for clear guidelines on assessing the capacity of people with SMI to sign end‐of‐life documents, standardised templates for use across settings, and a compiled legal/ethical casebook to guide service users, family members, practitioners, and the public. Expanding low‐cost or publicly funded legal services—potentially through partnerships with law schools, governmental and non‐governmental organisations—was proposed to improve access. To combat stigma and taboos, culturally sensitive life‐and‐death education and mental health campaigns were recommended, highlighting the rights and needs of people with SMI at the end of life. Developing supported decision‐making frameworks tailored to local legal and cultural norms was also proposed by several participants as essential for ensuring that service users can make informed choices, with reference to successful adaptations in other countries such as Australia and the UK. Finally, participants encouraged mobilising community involvement through developing dedicated volunteer training programs to ensure that palliative and end‐of‐life care for people with SMI gains the same visibility, recognition, and support as eldercare services.

## 4 Discussion

Drawing on stakeholder perspectives, this co‐design study offers a comprehensive view of the current state of end‐of‐life care for people with SMI in Hong Kong, the key challenges in its provision, and the preferred solutions. In general, our findings show some alignment with observations in the international literature on palliative and end‐of‐life care for this group. Meanwhile, they reveal several important insights that merit our attention, which highlight the complex intersective effects between mental health, death‐related issues, and Hong Kong's cultural and practice contexts.

While much of the literature emphasises the reciprocal impacts of mental and physical symptoms of terminal illness, as well as medication side effects, in end‐of‐life care (McNamara et al. 2018; Morgan and Coyle 2016), participants in this study rarely placed emphasis on these issues. Even when researchers presented these findings during the literature review session prior to group discussions or intentionally inquired about them in consultations, few relevant data or observations emerged. This absence of discussion should not be interpreted as suggesting that these impacts are unimportant in care provision. On the contrary, it is more likely to reflect that most professionals supporting clients in residential or community settings have neither the opportunity nor the awareness to observe such interactions in their clinical practice. Moreover, the JCECC: Unison clinical teams did not have long‐term, in‐depth engagement with clients, making it difficult for them to identify and compare the effects of these impacts. These findings underscore the importance of on‐site end‐of‐life care training and clinical supervision to strengthen practitioners' capacity to recognise and respond to the interplay of mental and physical health factors in practice.

Throughout the consultations and workshop discussions, one of the most consistent themes was the difficulty of engaging in death‐related conversations, whether from the perspective of people with SMI or practitioners. Research in other regions has shown that such conversations are often problematised by a presumption of incapacity or fears that they may trigger mental instability (Appelbaum 2007; Candilis et al. 2004). In Hong Kong, this challenge is magnified by a prevailing death‐denial culture and a stigmatising environment for people with SMI. In Chinese culture, death is widely regarded as a taboo subject, often avoided for fear of attracting bad luck (Tu et al. 2022). This cultural context helps to explain why concerns were raised by other service users sharing the same facilities, some of whom objected to the establishment of end‐of‐life care spaces, and why there is a pressing need for educational initiatives to cultivate a compassionate culture that supports people with terminal illness. Beyond cultural influences, the impact of stigmatising stereotypes should not be underestimated. While recent studies highlight the capacity of active participation of people with SMI in making end‐of‐life decisions (Relyea et al. 2019; Shalev et al. 2020), in Hong Kong, discussions about death, or even topics hinting at death, in the mental health context, are often quickly associated with fears of mental distress, relapse, or suicidal ideation. This stems not only from stereotypes about SMI but is also exacerbated by an entrenched risk‐averse culture in the mental health sector, where perceived ‘critical risks’ are linked to concerns about professional accountability (Cui et al. 2021). These findings underscore the importance of professional education that permeates all staffing levels, from frontline workers to managerial staff.

Furthermore, the influence of familial culture on end‐of‐life care may present different patterns for people with SMI compared with the general older population. In Chinese culture, filial piety and strong family bonds are deeply ingrained values. Family involvement in end‐of‐life care is often extensive and influential; in some cases, filial devotion has been reported to drive the persistent pursuit of innovative treatments, sometimes bypassing the patient's own decisions (Ohs et al. 2015). However, our findings showed that many people ageing with SMI tend to have relatively limited or distant personal and family relationships, whether living in the community or in residential settings—a trend similarly noted in previous research (Hannigan et al. 2022; Woods et al. 2008). This disparity can create a sharp contrast in the resources available to support end‐of‐life care compared with care models implemented for the general elderly population, underscoring the need for interventions tailored to those without family support or in unsupportive environments in mental health contexts. This, again, highlights the importance of clear guidelines and legal protections to safeguard the autonomy and dignity of people with SMI, and to prevent potential exploitation through fraud or unethical practices in the absence of family support during the final stages of life. In addition, another underutilised resource—though not emphasised by participants—is peer support, which has become an important element of mental health care (Joo et al. 2022). Co‐residents or members of mental health peer groups, some of whom may have known the individual for many years, could be mobilised to provide essential emotional and social support in their final stage of life.

Last but not least, in developing specialised care models for people with SMI, it is important to tailor the service according to individuals' needs because the users can be highly heterogeneous. One example is the wide variation in their levels of cognitive capacity. Accordingly, person‐centredness should be reflected in practice to accommodate diverse needs. This also points to a limitation of the present study. Although previous research has shown that people with different SMI diagnoses may face unique challenges during end‐of‐life care (Hinrichs et al. 2022), our study did not examine these distinctions in depth, as the project remains in its initial phase. Differences in end‐of‐life support needs by severity and diagnostic type (e.g., psychosis vs. mood disorders) may warrant further exploration in future research and clinical reporting.

## 5 Conclusion

Using a co‐design approach, this paper synthesised insights from a literature review, clinical consultations, and co‐design workshops on the development of end‐of‐life care for people with SMI. Findings revealed the suboptimal state of such care, shaped by barriers at individual, organisational, and community levels, alongside a range of co‐developed recommendations for reasonable adjustments to the existing system. Importantly, the paper highlights the value of involving multiple stakeholders—especially people with SMI—in the design and refinement of end‐of‐life care. Furthermore, as existing literature in this area is largely grounded in Anglophone and European contexts, this study, situated in a setting shaped by traditional cultural values surrounding death and mental illness, offers valuable insights into the intersecting sociocultural dimensions of mental health and end‐of‐life care.

## 6 Relevance to Clinical Practice

This paper directs attention to end‐of‐life care for people with SMI, an area that remains relatively underexplored, where practitioners such as nurses and social workers in mental health and palliative care often lack holistic, intersecting knowledge and awareness of the collaboration required in care delivery. The study resulted in a set of co‐designed recommendations, shaped by the local cultural, political and service contexts, spanning individual, organisational and community levels, highlighting priorities such as the development of tailored assessment tools, supported decision‐making frameworks, stronger intersectoral collaboration, clear guidelines for dying in place and culturally sensitive education and stigma reduction campaigns. The next step is to work closely with service users, policymakers and community partners to translate these recommendations into targeted, culturally responsive interventions that can be implemented, tested and sustained within routine practice, ultimately improving the quality and equity of care for this underserved population.

## Funding

This work was supported by The Hong Kong Jockey Club Charities Trust (grant number: 2024‐0088‐011).

## Conflicts of Interest

The authors declare no conflicts of interest.

## Data Availability

The data that support the findings of this study are available on request from the corresponding author. The data are not publicly available due to privacy or ethical restrictions.
